# Neural Autoantibodies in Cerebrospinal Fluid and Serum in Clinical High Risk for Psychosis, First-Episode Psychosis, and Healthy Volunteers

**DOI:** 10.3389/fpsyt.2021.654602

**Published:** 2021-03-26

**Authors:** Christian G. Bien, Cathrin Rohleder, Juliane K. Mueller, Corinna I. Bien, Dagmar Koethe, F. Markus Leweke

**Affiliations:** ^1^Department of Epileptology (Krankenhaus Mara), Medical School, Bielefeld University, Bielefeld, Germany; ^2^Laboratory Krone, Bad Salzuflen, Germany; ^3^Brain and Mind Centre, Faculty of Medicine and Health, The University of Sydney, Sydney, NSW, Australia; ^4^Department of Psychiatry and Psychotherapy, Medical Faculty Mannheim, Central Institute of Mental Health, Heidelberg University, Mannheim, Germany; ^5^Department of Psychiatry, Psychosomatics, and Psychotherapy, Goethe University, Frankfurt, Germany; ^6^Sydney Local Health District, New South Wales Health, Sydney, NSW, Australia

**Keywords:** autoimmune-mediated psychosis, autoimmune encephalitis, anti-neural autoantibodies, at-risk mental state, clinical high at-risk mental state, ultra-high risk for psychosis, schizophrenia, healthy control

## Abstract

The pathophysiological role of neural autoantibodies in acute psychotic disorders is receiving increased attention. However, there is still an ongoing debate, whether predominantly psychotic manifestations of autoimmune encephalitides exist that may remain undetected and, thus, untreated. Furthermore, it is discussed if such conditions can be diagnosed based on serum antibody results or if a reliable diagnosis requires additional cerebrospinal fluids (CSF) results. In this study, we screened pairs of serum and CSF samples from antipsychotic-naïve individuals with first-episode schizophrenic psychosis (FEP, *n* = 103), clinical high risk for psychosis (CHR, *n* = 47), and healthy volunteers (HV, *n* = 40) for eight different antibodies against various antigens that have been shown to be associated with autoimmune encephalitides: N-methyl-D-aspartate receptor (NMDAR, NR1 subunits only), glutamic acid decarboxylase (GAD65), leucine-rich glioma inactivated protein 1 (LGI1), contactin-associated protein-like 2 protein (CASPR2), α-amino-3-hydroxy-5-methyl-4-isoxazolepropionic acid receptor (AMPAR) subunit 1, AMPAR subunit 2, γ-aminobutyric acid-B receptors (GABA_B_R), and glycine receptors. All patients were within the norm with regards to a careful neurological examination, a magnetic resonance imaging (MRI) of the brain, an electroencephalogram (EEG), and routine blood pathology. All CSF samples were autoantibody-negative. In three serum samples of individuals with FEP, we detected low-titer CASPR2 immunoglobulin (Ig) G antibodies (≤1:160, *n* = 2) and non-IgG antibodies against NMDAR (*n* = 1) (overall serum-autoantibody prevalence in FEP: 2.91%). However, the IgG titers were below the laboratory cut-off defined for positivity, and non-IgG antibodies are of no clinical relevance. This suggests that there were no cases of autoimmune encephalitis in our cohort. Our results highlight the importance and the high specificity of CSF analysis to reliably detect autoantibodies. They confirm the hypothesis that pure psychotic manifestations of antibody-associated autoimmune encephalitides without any additional neuropsychiatric findings are very rare. However, special attention must be paid to those presenting with atypical mental illnesses with additional neurological symptoms, evidence of clinically-significant cognitive involvement, profound sleep-wake perturbations, seizures, electroencephalographic, or magnetic resonance imaging pathologies to be able to identify cases with autoimmune-mediated psychiatric syndromes.

## Introduction

The autoimmune encephalitides are defined by immunoglobulin (Ig) G autoantibodies against neural antigens (including cell surface antigens; intracellular, onconeural antigens; and intracellular, synaptic antigens) and are clinically characterized by transdiagnostic neurological and psychiatric symptoms (1). The leading example in terms of frequency, disease severity, psychotic symptoms, and treatment responsivity is anti-N-methyl-D-aspartate receptor (NMDAR) encephalitis. There is an ongoing debate, whether pure psychotic cases exist as *formes frustes* of autoimmune encephalitides, which might potentially respond as well to immunotherapy as the full-blown forms. As Susannah Cahalan put it in her famous book about her own anti-NMDAR encephalitis: “If it took so long for one of the best hospitals in the world [to make this diagnosis], how many other people were going untreated, diagnosed with a mental illness or condemned to a life in a nursing home or a psychiatric ward?” (2).

Experts in the field have emphasized that isolated psychiatric (predominantly psychotic) manifestations in definite autoimmune encephalitides are rare, e.g., only 4% in a sample of 571 cases with autoimmune encephalitis and NMDAR antibodies in cerebrospinal fluid (CSF) (3). Consequently, such experts suggested being vigilant for patients with psychoses plus other neurological or cognitive problems, all emerging in a subacute fashion (4). Meanwhile, international criteria for the clinical diagnosis of an “autoimmune psychosis” have been suggested to improve the recognition of psychosis of suspected autoimmune origin (5). Furthermore, experiences of immunomodulating therapy attempts and therapeutic considerations have recently been published (6, 7).

Several groups studied series of patients with psychosis, often first-episode psychosis (FEP) without predominant neurological features, mostly in serum, and always found some autoantibody-positive patients; the positive results have recently been reviewed and summarized (8). One group studied sera of patients with schizophrenia and controls for neural autoantibodies. IgG autoantibodies were found at frequencies that were in the range of healthy controls; more frequently, non-IgG antibodies were detected, again at similar percentages as in controls (e.g., NMDAR antibodies—IgG/IgM/IgA: schizophrenia 0.6%/5.0%/5.3% and healthy controls 1.2%/4.3%/4.5%, respectively). Antibody titers in the psychiatric and healthy individuals were ≤1:320, i.e., low (9). These data can be interpreted in two ways: The senior author of this study argued that the antibodies, together with additional abnormalities like blood-brain barrier leakiness, might be causative for psychoses (10, 11). Others concluded that these low-titer IgG antibodies in serum were non-specific and irrelevant or even potentially false-positives (12). Another study reported a prevalence of serum antibodies against neural cell surface antigens of 9% among 228 people presenting with FEP and 4% among 105 healthy controls, whereby NMDAR antibodies (IgG) were detected in 3% of FEP cases but in none of the healthy controls (13). While some experts feel confident in diagnosing “autoimmune psychosis” based on serum antibody results (5, 13), others keep asking for CSF results—some for concern of specificity of serum-only findings (14), others because antibodies might be hidden in CSF and might go undetected by testing serum only—which is a variant of Susannah Cahalan's concern (2). Indeed, CSF-only reactivity has been shown for NMDAR antibodies in 14–28% of cases (15, 16).

To clarify these issues, neural antibodies in serum and CSF should be examined in larger numbers of psychiatric patients (17). Ideally, such a study would include healthy controls to provide information on background frequency in the population. Here, we provide such a study. We screened the serum and CSF from antipsychotic-naïve people with FEP or Clinically High Risk (CHR) states for psychosis and healthy volunteers for various antibodies that have been shown to be associated with autoimmune encephalitis.

## Methods

### Participants

Participants (*n* = 190) were recruited from two clinical sites with full community service obligations providing both inpatient units and outpatient services, including early recognition for psychosis units in Germany: The Department of Psychiatry and Psychotherapy, University of Cologne and the Department of Psychiatry and Psychotherapy, Central Institute of Mental Health (CIMH), Mannheim. The study protocol was approved by the Ethics Committee of the University of Cologne and the Ethics Committee II of the Medical Faculty Mannheim, Heidelberg University. All study participants provided written informed consent. Notably, the approved study protocol allowed for retrospective consent to the use of remaining CSF and serum samples obtained during a clinical CSF investigation, where individuals were able to consent to a CSF investigation prior to a lumbar puncture but could initially not consent to the research study. Procedures were performed in accordance with the code of ethics of the world medical association for experiments involving humans (Declaration of Helsinki).

Healthy volunteers (HV; *n* = 40, all from Cologne) had no history of mental illness and were recruited within the same geographic area and population. Individuals with CHR states (*n* = 47, 40 from Cologne, seven from Mannheim) met the following criteria: (1) attenuated positive symptoms, (2) brief, limited intermittent psychotic symptoms that spontaneously resolved within 1 week, and (3) a recent decline in function for at least 1 month, in combination with the existence of a first or second degree relative with a history of any DSM-IV (Diagnostic and Statistical Manual of Mental Disorders, fourth edition) psychotic disorder or met the criteria for schizotypal personality disorder. Individuals defined as having FEP (*n* = 103, 100 from Cologne, three from Mannheim) met the DSM-IV criteria for schizophrenia. All individuals with FEP were antipsychotic-naïve, but some individuals with either FEP or CHR received low-dose benzodiazepines (lorazepam, oxazepam, or diazepam). Noteworthy, patients were only included in this study when a careful neurological examination, a magnetic resonance imaging (MRI) of the brain, an electroencephalogram (EEG), and a routine blood pathology were within the norm as recommended by several guidelines [e.g., (18)].

Demographic and clinical information on age, gender, ethnicity, years of education, and symptom severity [i.e., Positive and Negative Syndrome Scale (PANSS) scores, assessed on the day of lumbar puncture] is given in Table 1. The three groups were comparable for ethnicity using 4 × 3 Fisher's exact test (*X*^2^= 4.4; *P* = 0.550). The majority of participants were Caucasian, but eight participants were non-Caucasian (one healthy control and seven people with schizophrenia). However, the groups differed significantly regarding age [analysis of variance (ANOVA), *F*_(2, 187)_ = 9.2; *P* ≤ 0.001]. As expected, Bonferroni *post hoc* tests revealed that individuals with CHR were significantly younger than individuals with FEP (*P* ≤ 0.001) but also than HV (*P* = 0.011). Furthermore, the proportion of males was higher among the individuals with CHR compared to FEP or HV [2 × 3 Fisher's exact test (*X*^2^= 8.7; *P* = 0.012) and subsequent z-test]. Individuals with CHR and FEP completed significantly fewer years of education than HV (3 × 3 Fisher's exact test (*X*^2^= 22.3; *P* ≤ 0.001) and subsequent z-test), possibly due to a need for treatment during illness.

**Table 1:** Demographic and clinical information of the study cohort.

	**HV**	**CHR**	**FEP**	***P*-value^a^**
*N*	40	47	103	
Age, years (mean ± SD [min, max])	28.6 ± 8.9 [18, 58]	23.3 ± 4.6 [17, 35]	29.5 ± 9.3 [16, 69]	≤0.001
Gender (male/female)	25/15	38/9	58/45	0.012
Ethnicity^b^	39/1/0/0	47/0/0/0	96/3/3/1	0.550
Education^c^ (≥13 years/ <13 years/unknown)	32/6/2	24/23/0	43/51/9	≤0.001
Antipsychotic medication (%)	0	0	0	
PANSS total (median [min, 25th, 75th, max], n)	–	70 [36, 55.75, 83.25, 104], 38	93 [45, 75.5, 107.5, 143], 97	–
PANSS positive (median [min, 25th, 75th, max], n)	–	15.5 [8, 12, 18, 24], 38	21 [10, 18, 26.5, 38], 97	–
PANSS negative (median [min, 25th, 75th, max], n)	–	17.5 [7, 11, 21, 32], 38	24 [9, 17, 29, 40], 97	–
PANSS general (median [min, 25th, 75th, max], n)	–	36 [21, 29, 44, 54], 38	48 [24, 38.5, 54, 73], 97	–

a *ANOVA or Fisher's Exact Test*.

b *Caucasian/African/Asian/Other*.

c *percentage that completed at least 13 years, a high-school diploma equivalent*.

The median PANSS total scores of 93 in the FEP group and 70 in the CHR group were within the expected range of symptom severity in acute psychotic conditions for FEP and CHR individuals, with the latter not fulfilling diagnostic criteria for schizophrenia but presenting with substantial, mostly intermittent symptoms. As far as retrospectively assessable, the average duration of illness did only slightly differ from the expected range (19), indicating the lower threshold of service supply by both centers. However, the respective duration showed a high variability ranging from several weeks to several years of retrospectively accessible psychotic symptoms.

All participants underwent lumbar puncture and venipuncture for the collection of CSF–serum pairs. Serum and CSF samples were stored at −80°C until being shipped to the Epilepsy Center Bethel, Krankenhaus Mara, Bielefeld, Germany.

### Antibody Screening

At the Epilepsy Center Bethel, all samples were stored at −20°C until testing with commercially available biochips (Euroimmun, Lübeck, Germany). These fixed cell-based assays (CBA) consist of human embryonic kidney (HEK 293) cells transfected with plasmids encoding the following antigens (fixation method in brackets): NMDAR consisting of NR1 subunits only, glutamic acid decarboxylase (GAD65, acetone), leucine-rich glioma inactivated protein 1 (LGI1), contactin-associated protein-like 2 (CASPR2), α-amino-3-hydroxy-5-methyl-4-isoxazolepropionic acid receptor (AMPAR) subunit 1, AMPAR subunit 2, γ-aminobutyric acid-B receptors (GABA_B_R), glycine receptors (paraformaldehyde). Their preparation follows an established method (20) and has been described in detail before (21).

Screening for antibodies was done in the Antibody Laboratory in Bethel, Bielefeld, Germany, following the manufacturer's recommendations (FA 112d-1005-1, IgG) with modifications (CGB); the datasheet instructions are given in brackets: serum samples were diluted to 1:16 as the screening dilution (Euroimmun: 1:10); buffer was PBS (Euroimmun: PBS-Tween); secondary antibody: goat-anti-human IgG (heavy and light chain [H+L]) conjugated with Alexa Fluor® 594 produced by Jackson ImmunoResearch, West Grove, PA, USA, Code No. 109-585-088, used at a dilution of 1:100, incubation 30 min at room temperature (RT) (Euroimmun: goat-anti-human IgG, conjugated with fluorescein, no further information given); nuclear counterstaining with Hoechst 33342, 1:10,000 (Euroimmun: no nuclear staining); embedding with 1,4-Diazabicyclo[2.2.2]octan (Euroimmun: glycerol). Stained biochips were examined using a fluorescence microscope (Leica DM 2000; Wetzlar, Germany) equipped with adequate filters. One of two neurologists experienced in reading this assay (CGB, CIB) decided if an antibody was present using the signal of the surrounding (supposedly negative) fields as respective negative controls. The readers were blind to the participants' diagnosis. The codes were only opened when all analyses had been completed and the manuscript draft was written.

Samples that reacted with the H+L secondary antibody were re-tested in the Laboratory Krone (after the facilities had been moved from The Mara to this lab in January 2016) by using a goat-anti-human antibody against the Fcγ fragment of IgG (conjugation and manufacturer as with H+L antibody, catalog no. 304-585-008) at a dilution of 1:100, incubation 30 min at RT. Samples that were not reactive with the Fcγ antibody or gave a much weaker Fcγ than H+L signal were investigated with specific IgA secondary antibodies (dianova, catalog no. 109-585-129, 1:100, 30 min at RT, directly labeled with Alexa 594) and IgM secondary antibodies to clarify this discrepancy (dianova, catalog no. 109-545-129, 1:100, 30 min at RT, labeled with Alexa 488 H+L). H+L antibodies also bind to high titer IgA and IgM antibodies, whereas IgG-Fcγ antibodies are IgG specific but may not be as sensitive as the H+L antibodies. True IgG antibodies were endpoint titrated with the IgG-Fcγ antibody in multiples of 1:2 and rated by two investigators (CGB, CIB, or experienced technician) independently. In cases of divergent ratings, the mean of the two ratings was recorded. The titration steps for serum were: 1:20, 1:40, 1:80, and so forth; CSF: neat, 1:2, 1:4, and so forth. In general, IgG antibodies were considered positive if serum titers were ≥1:16 or were found in CSF at any dilution starting from neat. For anti-CASPR2-encephalitides, this lab determined a serum titer cut-off of ≥1:512 for MRI-negative cases as included in this study (22).

Sera were also tested for onconeural antibodies (anti-HuD, anti-Yo, anti-Ri, anti-CV2, anti-Amphiphysin, anti-Ma 1, anti-Ma2, anti-SOX1) by an immuno-dot-blot according to the manufacturer's recommendation (Ravo Diagnostika, Freiburg, Germany).

## Results

In this study on individuals with CHR or FEP and HV, three individuals harbored neural antibodies. These were only low-titer (22) CASPR2 serum IgG antibodies (≤1:160) or non-IgG antibodies against the NMDAR or CASPR2. They were found in three out of 103 participants with FEP [prevalence: 2.91% (overall)/1,94% (CASPR2 IgG)/0.97% (NMDAR non-IgG) details see Tables 2, 3].

**Table 2:** List of neural autoantibody-positive samples.

**Study ID**	**CSF/Serum**	**IgG**	**IgA**	**IgM**
689	Serum	CASPR2 1:160	Negative	Negative
331	Serum	CASPR2 1:20	Negative	CASPR2 1:320
542	Serum	Negative	NMDAR 1:640	NMDAR 1:640

*CSF, cerebrospinal fluid; Ig, immunoglobulin; CASPR2, contactin-associated protein-2; NMDAR, N-methyl-D-aspartate receptor*.

**Table 3:** Prevalence of neural autoantibodies in serum and CSF of individuals with CHR states or schizophrenia and healthy controls.

	**Titers (serum/CSF)**	**HV (serum/CSF)**	**CHR state (serum/CSF)**	**FEP (serum/CSF)**
*N*		40/40	47/47	103/103
NMDAR (NR1 subunit)	1:640^a^/–	0/0	0/0	1 (0.97%)^a^/0
GAD65	–/–	0/0	0/0	0/0
LGI1	–/–	0/0	0/0	0/0
CASPR2	1:20–1:160^b^/ –	0/0	0/0	2^b^ (1.94%)/0
AMPAR subunit 1	–/–	0/0	0/0	0/0
AMPAR subunit 2	–/–	0/0	0/0	0/0
GABA_B_R	–/–	0/0	0/0	0/0
Glycine receptors	–/–	0/0	0/0	0/0

a *non-IgG antibodies*.

b *IgG antibodies*.

*AMPAR, α-amino-3-hydroxy-5-methyl-4-isoxazolepropionic acid receptor; CASPR2, contactin-associated protein-like 2; CSF, cerebrospinal fluid; GABA_B_R, γ-aminobutyric acid-B receptors; GAD65, glutamic acid decarboxylase 65 kDa; LGI1, leucine-rich glioma inactivated protein 1; NMDAR, N-methyl-D-aspartate receptor*.

None of the CSF samples, neither from individuals with CHR or FEP nor HV, harbored neural autoantibodies (i.e., prevalence of 0% in each group).

## Discussion

This series of serum-CSF pairs of individuals with CHR or FEP and HV did not reveal CSF antibodies against a broad panel of neural intracellular and surface antigens, including the NMDAR. There were only two cases with serum IgG antibodies. Both were CASPR2 antibodies in serum only. Their titers were below the cut-off defined for positivity for autoimmune encephalitides in this laboratory (22), suggesting that they are no cases of autoimmune encephalitis. The absence of CSF antibodies in this series supports the notion of the high specificity of neural antibodies detected in that compartment. It also shows that there are probably not “many” people with antibody-associated autoimmune encephalitides in a psychiatric institution as Susannah Cahalan was worrying (2)—at least not among the cohort who contributed to this study and were aged between 16 and 69 years.

The study was not explicitly designed to identify IgA and IgM antibodies. In two serum samples, such antibodies were found when clarifying a mismatch of the non-IgG specific H+L secondary antibody and the IgG specific secondary antibody against the Fcγ fragment. These were likely the highest titer non-IgG antibodies in this series. Lower titer IgA or IgM antibodies may have gone undetected by the H+L secondary antibodies. This means there may be more non-IgG antibody positives in this series not revealed by our approach. Of note, non-IgG antibodies against the NMDAR have been found to be non-specific (23).

Within a series of 121 people with the initial diagnosis of schizophrenia, one study finally found two individuals with antibodies against the NR1 subunit of the NMDAR in CSF—both were re-classified as anti-NMDAR encephalitis because they had the characteristic polymorphic presentation (24). Another study (25) did serial serum and CSF antibody tests in 113 individuals admitted with an FEP; they found one without prominent neurological features and NMDAR antibodies in CSF (0.9%). Two other participants with such antibodies developed complex encephalopathies, had ovarian teratomata, and were, therefore, typical for anti-NMDAR encephalitis (25).

Apart from these cases, there have been several reports of patients with NMDAR antibodies in serum but not in CSF or of non-IgG NMDAR antibodies—both constellations are not disease-specific (15, 23). One reason for false-positive serum results is a misinterpretation of staining signals (26–28). As an alternative explanation, it has been suggested that the brain might “immunoprecipitate” NMDAR antibodies from the CSF, which would lead to serum-positive/CSF-negative constellations (29).

A recent meta-analysis found that the serum NMDAR IgG antibody prevalence in individuals with psychosis is associated with the assay type. More precisely, compared to a fixed CBA, a live CBA was associated with a significantly increased prevalence and significantly higher odds ratios when comparing patients and controls (30). The authors concluded that the live CBA leads to improved detection of NMDAR IgG antibodies in serum, but that studies directly comparing both detection methods are needed to confirm this finding. One study (31) re-tested 14 NMDAR IgG antibody-positive serum samples identified by a live CBA among 298 serum samples of individuals with FEP in two different laboratories using a live or fixed CBA, respectively. The fixed CBA detected only one positive serum and one weakly positive serum among the 14 samples, while the live CBA detected nine out of 14 positive samples. A more systematic study confirmed that live CBAs are more sensitive with serum testing than fixed CBA, however, at the price of a reduced specificity (32). A lower specificity of the serum live CBA has already been described by the Barcelona/Philadelphia groups (15). It has been commonly agreed that CSF positivity is the decisive factor in diagnosing autoimmune encephalitis. The advantage of the present study is that CSF-serum pairs were available for all individuals included. The high sensitivity and specificity of CSF test results with fixed CBA are beyond discussion (15).

Notably, the individuals with CHR and FEP included in this study were carefully assessed and fulfilled not only DSM-IV criteria but were also found to be within the normal range regarding a careful neurological examination, a brain MRI, an EEG, and extensive blood pathology. Therefore, our study results particularly highlight that isolated, psychotic manifestations of autoimmune encephalitides occur only sporadically. That is, clinicians need to be specifically vigilant when individuals present with additional neurological symptoms, evidence of clinically-significant cognitive involvement, profound sleep-wake perturbations, EEG or brain MRI pathologies (5). Experts in the field have suggested the following features that should lead to EEG diagnostics, lumbar puncture, and serum and CSF diagnostics for neural autoantibodies: memory deficits/disorientation, disorganized-bizarre behavior, stereotypia or catatonia, dyskinesias, and dysphagia, or autonomic dysfunction (33). Such recommendations have been implemented in the German S3-Schizophrenia guidelines. It lists “hard signs“ (CSF pleocytosis, epileptic seizures, faciobrachial dystonic seizures, mediotemporal MRI lesions, and EEG abnormalities) and “soft signs” (loss of vigilance, movement disorder of unsteady gait and stance, autonomic instability, focal neurological deficits like aphasia or dysarthria, rapid progression of psychotic signs and symptoms despite treatment; hyponatremia, catatonia, unexplained headache, comorbid autoimmune disorders) for the suspicion of autoimmune encephalitis underlying a psychotic presentation. Referencing the international “clinical approach to the diagnosis of autoimmune encephalitis,” they recommend MRI and CSF studies, including neural antibodies (34).

### Limitations

Since none of the healthy controls was younger than 18 years, these findings are not valid for pediatric and geriatric populations. Furthermore, despite a good clinical characterization of the participants presenting to two different clinical sites affiliated with universities, the overall sample size (*n* = 190) is still relatively small to draw general conclusions on the relevance of the neural autoantibodies in general psychiatry settings.

The prevalence of neural antibodies may be higher among those individuals with FEP that are most unwell. For instance, Scott et al. (25) reported that five out of six antibody-positive patients were too unwell to initially consent, requiring an alternative decision-maker to enable participation. Therefore, it is nearly impossible to avoid a certain selection bias in research studies requiring written informed consent. In our study, the enrolled individuals with FEP were severely ill (as indicated by the median PANSS total score of 93). However, the approved study protocol allowed for retrospective consent to the use of remaining CSF and serum samples obtained during a clinical CSF investigation, where consent to a CSF investigation was feasible prior to performance of a lumbar puncture, but no consent was possible to the study informed consent procedure. Thus, the data is based on a representative sample of patients from two full community-serving hospitals, treating patients throughout the full spectrum of symptoms severity.

As this study focused on neural autoantibodies, we did not screen for elevated antibody levels against thyroid antigens [thyroperoxidase (TPO), thyroglobulin (TG), thyrotropin (TSH) receptor]. However, these systemic non-CNS (central nervous system) autoantibodies have also been reported to be associated with neuropsychiatric manifestations (35).

Furthermore, we did not include samples from individuals with atypical psychotic syndromes. Future studies should compare the prevalence of neural autoantibodies in serum and CSF samples of individuals with schizophrenia, atypical psychotic syndromes, or atypical mood syndromes, and healthy controls to better understand the complex clinical picture of autoimmune-mediated psychiatric syndromes.

## Conclusion

In summary, our study speaks against a major role of neural antibody-associated autoimmune encephalitides in the conditions studied here, namely typical presentations of CHR and FEP. However, there is emerging evidence of the involvement of various neural autoantibodies and systemic non-CNS autoantibodies in *atypical* psychotic and mood syndromes. Therefore, it is essential to pay attention to those who present with polymorphic psychiatric symptoms, significant cognitive involvement, and neurological phenomena in daily clinical practice. Further, performing a careful neurological examination as well as a brain MRI scan and an EEG is essential to not miss neural antibody-associated autoimmune conditions. Consequently, in line with the majority of international guidelines, a comprehensive assessment is needed to detect autoimmune encephalitides. Our study highlights the importance of CSF analysis to reliably detect the involvement of immune processes and demonstrates the high specificity of positive CSF test results by the absence of detectable antibodies from our healthy controls.

## Data Availability Statement

The raw data supporting the conclusions of this article will be made available by the authors, without undue reservation.

## Ethics Statement

The studies involving human participants were reviewed and approved by Ethics Committee of the University of Cologne and Ethics Committee II of the Medical Faculty Mannheim, Heidelberg University. The patients/participants provided their written informed consent to participate in this study.

## Author Contributions

FML, CGB, and CR conceived and designed the study. FML, JKM, and DK enrolled the participants and obtained data and body fluids. CGB and CIB analyzed the CSF and serum samples and the data. CGB, FML, and CR drafted the manuscript. All authors contributed to the final manuscript preparation and have read and approved the final manuscript.

## Conflict of Interest

FML was a shareholder of curantis UG (Ltd.) and receives research support from the German Federal Ministry of Education and Research (BMBF), the Moyira Elizabeth Vine Fund for Research into Schizophrenia, and the DVCR research fund of the University of Sydney. CGB receives research support from the Deutsche Forschungsgemeinschaft (German Research Council, Bonn, Germany) and Gerd-Altenhof-Stiftung (Deutsches Stiftungs-Zentrum, Essen, Germany). The remaining authors declare that the research was conducted in the absence of any commercial or financial relationships that could be construed as a potential conflict of interest.
